# *Salvia miltiorrhiza* Protects Endothelial Dysfunction against Mitochondrial Oxidative Stress

**DOI:** 10.3390/life11111257

**Published:** 2021-11-18

**Authors:** Yu-Chen Cheng, I-Ling Hung, Yen-Nung Liao, Wen-Long Hu, Yu-Chiang Hung

**Affiliations:** 1 Department of Chinese Medicine, Kaohsiung Chang Gung Memorial Hospital and Chang Gung University College of Medicine, Kaohsiung 83342, Taiwan; ycc0504@cgmh.org.tw (Y.-C.C.); izzyizzy@cgmh.org.tw (I.-L.H.); lyn6655@adm.cgmh.org.tw (Y.-N.L.); oolonghu@cgmh.org.tw (W.-L.H.); 2 Graduate Institute of Clinical Medical Sciences, College of Medicine, Chang Gung University, Kaohsiung 33302, Taiwan; 3 College of Medicine, Kaohsiung Medical University, Kaohsiung 80708, Taiwan; 4 College of Nursing, Fooyin University, Kaohsiung 83102, Taiwan

**Keywords:** *Salvia miltiorrhiza*, mitochondria, endothelial dysfunction, oxidative stress, TCM, herbal medicine, reactive oxygen species, cardiovascular disease

## Abstract

*Salvia miltiorrhiza* (SM) is a common traditional Chinese medicine used in the treatment of cardiovascular and cerebrovascular diseases. Endothelial dysfunction plays an important role in the pathology of cardiovascular diseases. Endothelial dysfunction may induce inflammation and change vascular tone and permeability. The main pathological mechanism of endothelial dysfunction is the formation of reactive oxygen species (ROS). Mitochondria are the main source of energy and can also produce large amounts of ROS. Recent studies have shown that extracts of SM have antioxidative, anti-inflammatory, and antithrombus properties. In this review, we discuss the mechanism of oxidative stress in the mitochondria, endothelial dysfunction, and the role of SM in these oxidative events.

## 1. Introduction

### 1.1. Reactive Oxygen Species and Oxidative Stress

Free radicals may damage human health. There are many oxidative agents in our environment (air, water, tobacco, alcohol, heavy or transition metals, drugs, industrial solvents, radiation, etc.) and also produced inside the human body. Oxidative stress occurs due to oxygen-related metabolic reactions. It originates from the disequilibrium between reactive oxygen species (ROS) formation and enzymatic and nonenzymatic antioxidants in living organisms [1].

ROS can be produced in compartments, such as the cytoplasm, mitochondria, peroxisomes, and endoplasmic reticulum [2]. While cells suffer from oxidative stress, ROS are produced from the respiratory chain, leading to electron transfer. The superoxide radical (O_2_^•−^), which dismutates from hydrogen peroxide (H_2_O_2_) and molecular oxygen (O_2_), is a toxic compound following ROS stimulation [3,4]. Oxidative stress causes cell damage through three mechanisms: lipid peroxidation of membranes, oxidative modification of proteins, and DNA damage [5]. In the challenge of physical stresses like genotoxic stress and viral infection, ROS and reactive nitrogen species (RNS), as the intracellular signal transducers, may lead to autophagy, which is a catabolic process by damaging organelles and recycling cellular components [6].

Because of this, the results might cause aging and many human diseases, such as cancer, cardiovascular disease, metabolic disease, and infectious disease [7].

### 1.2. Endothelium

The endothelium exists in blood vessels, lymphatic vessels, and cornea. In this review article, we mainly discuss the endothelium in blood vessels. The arterial vessel is outlined by three distinct layers. The first layer is the tunica intima, a single layer of squamous endothelial cells, covering the internal surface of vessels; the second layer is the tunica media, which comprises vascular smooth muscle cells (VSMCs); and the final layer is the tunica adventitia, an elastic lamina with terminal nerve fibers and surrounding connective tissue. The endothelium [8] plays an extensive variety of essential roles in the control of vascular function, not only as a barrier between blood and tissues but also as an endocrine organ. The functions of the endothelium are as follows: (1) maintaining the balance between coagulation and fibrinolysis to provide the proper hemostatic balance [9]; (2) regulating coagulation [10,11]; (3) platelet adhesion and aggregation; (4) inflammation [12], (5) leukocyte activation, adhesion, and transmigration [13,14]; (6) regulating the regional blood flow to maintain the vascular tone and growth [15]; and (7) control of cell proliferation and angiogenesis [16].

In the normal vascular endothelium, high levels of nitric oxide (NO) and prostacyclin (PGI_2_) [17] and low levels of ROS, uric acid, endothelin-1 (ET-1), and angiotensin II (Ang II) contribute to endothelium-dependent vasodilatation [18]. The levels of inflammation-related factors, such as C-reactive protein (CRP), tumor necrosis factor-α (TNF-α), interleukin-6 (IL-6), soluble intercellular adhesion molecule (sICAM), soluble vascular cell adhesion molecule (sVCAM), and E-selectin, are low in physical conditions as well. When it comes to the anticoagulative status in the endothelium, levels of von Willebrand factor (vWF), plasminogen activator inhibitor-1 (PAI-1), and P-selectin are low [19,20].

### 1.3. Mitochondria

Mitochondria are important for energy metabolism [21]. The mitochondrial respiratory chain, which takes place in the inner mitochondrial membrane, is vital for energy metabolism. It is made of five compounds (complex I, II, III, IV, and V) [22], which catalyze the phosphorylation of ADP to ATP by electron transfer between them [23]. In 1956, Denham Harman first mentioned that free radicals, as by-products of the normal metabolism of mitochondria, attack cell constituents [24]. The following studies also suggest that intracellular ROS production is caused by the mitochondria. The production of mitochondrial superoxide radicals occurs primarily at complex I (NADH) and complex III (ubiquinone-cytochrome c reductase) [25]. In complex I, the electron transport chain of NADH and FADH_2_ transfers electrons to oxygen and hydrogen, and ROS or RNS production might occur during these electron transport steps. Under the general metabolic status, complex III is the main place of ROS production [26].

Mitochondria are also involved in several cellular processes, such as signaling through mitochondrial ROS (mtROS) [27], regulation of calcium storage [28], steroid generation [29], cellular differentiation, mitophagy, and apoptosis [30]. These functions maintain and control the cell cycle, cell growth, and cell death. The cytosolic Ca^2+^, which is released by the endoplasmic reticulum, is internalized by mitochondria via the uniporter and released by Na^+^/Ca^2+^ or H^+^/Ca^2+^ exchangers [31]. In both the intermembrane space and the matrix of mitochondria, while Ca^2+^ is taken up, it regulates the activity of transporters, enzymes, and proteins involved in organelle metabolism [32]. Steroidogenesis is a multistep process for biosynthesis of steroid hormone from cholesterol. Mitochondria-associated endoplasmic reticulum membranes (MAMs) play important roles in regulating steriodogenesis and maintain many cellular functions, including lipid metabolism, calcium signaling, and apoptosis [33]. During the cell cycle, mitochondrial membrane permeabilization (MMP) is mainly the decisive event in cell death through the release of catabolic hydrolases and enzymes [30].

### 1.4. Salvia miltiorrhiza

*Salvia miltiorrhiza* (SM) belongs to the Lamiaceae family and is a traditional Chinese medicine (TCM), also known as Danshen, in its Chinese name. SM is widely used for the treatment of circulatory diseases, including cardiovascular and cerebrovascular diseases. It has multiple biological functions, such as antioxidative stress, anti-inflammation, and antithrombosis. Based on previous studies, there are more than 49 diterpenoid quinones, and over 36 water-soluble phenolic acids and 23 essential oil components have been identified and isolated from SM [34].

The predominant bioactive compounds in SM contain two major groups of chemicals. One group includes lipophilic compounds (terpenoids), such as tanshinone I (Tan I), tanshinone IIA (Tan IIA), acetyltanshinone IIA, cryptotanshinone, isocryptotanshinone, dihydrotanshinone, 15,16-dihydrotanshinone I, and miltirone. These terpenoids exhibit a spectrum of potential biological activities, including antioxidant, antibacterial, anti-inflammatory, antiatherogenic, neuroprotective, antitumor, and antidiabetic effects. The other group includes hydrophilic phenolic acids, such as caffeic acid, danshensu, salvianolic acid A (SalA), salvianolic acid B (SalB), lithospermic acid, and lithospermic acid B. These polyphenol structures protect the cardiovascular system via ROS scavenge, leukocyte–endothelial adherence reduction, aortic smooth muscle cell inflammation, and metalloproteinase expression inhibition, as well as competitive binding of salvianolic acids to target proteins to interrupt protein–protein interactions [35]. Our previous population-based studies also revealed that SM is the most commonly used TCM for ischemic heart disease [36] and ischemic stroke [37] treatment. Wang et al. [38] reviewed 39 clinical trials using SM treatment for cardiovascular diseases and the conclusions supported that SM had beneficial therapeutic properties for cardioprotective effects through different cell signaling pathways. Several reviews have also provided the same conclusions not only in cardiovascular disease [39,40] but also in metabolic syndrome patients [41].

Mitochondrial oxidative stress may lead to endothelial dysfunction, which is known to be associated with cardiovascular disease and aging. We review current studies in this field to show the mechanisms of SM in protecting endothelial dysfunction against mitochondrial oxidative stress.

## 2. Monograph of Mitochondrial Oxidative Stress, Endothelial Dysfunction, and *Salvia miltiorrhiza*

### 2.1. Mitochondrial Oxidative Stress

**Figure 1:**
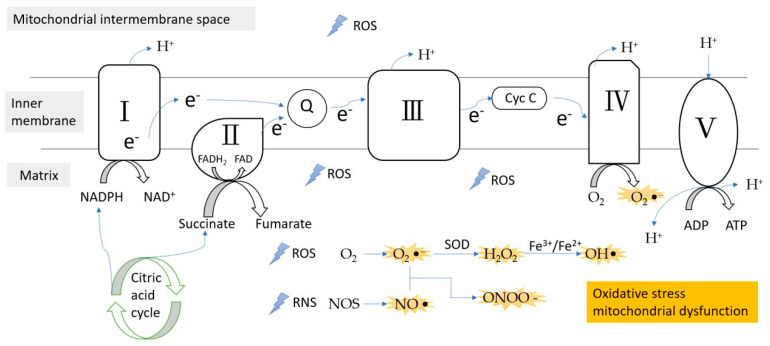
ROS/RNS formation during the electron transfer chain in the inner membrane of mitochondria. Complex III is the main site of ROS production. ROS, reactive oxygen species; RNS, reactive nitrogen species; e^−^, electron; Q, coenzyme Q; Cyc C, cytochrome c; SOD, superoxidase dismutase.

Mitochondria are one of the main sources of oxidative stress, as they use oxygen for energy production. ROS and RNS, generated by tightly regulated enzymes, are involved in many processes of normal physiology, such as signaling transferring pathways, induction of mitogenic response, and defense against pathogens. Excessive stimulation of NAD(P)H and the electron transport chain leads to the overproduction of ROS, which results in oxidative stress (Figure 1). Once oxidative stress is induced, it causes irreversible injury to proteins, lipids, and mitochondrial nucleic acid components, leading to the release of cytochrome c in the cytosol, which in turn causes apoptosis. These have been considered to be the main causes of many diseases, including neurodegenerative diseases [42], malignant tumor, ischemic heart disease, and diabetes [7].

Mitochondrial dysfunction triggered by any pathological situation can lead to a significant increase in ROS levels. For instance, hypoxia is the deficiency of oxygen, which is the terminal acceptor of electrons from the electron transport chain. Moreover, xenobiotics or their metabolic by-products can also lead to mitochondrial dysfunction through many kinds of mechanisms [43,44].

The basis of the free radial theory for aging in mitochondria is related to oxidative stress, which leads to mitochondrial DNA mutations. The accumulation of mitochondrial DNA mutations causes oxidative phosphorylation malfunction, and imbalanced antioxidant enzymes leads to overproduction of ROS and the formation of a vicious cycle [31]. Oxidative stress also deranges the mitochondrial respiratory chain, affects Ca^2+^ homeostasis [32,45], and influences membrane permeability and mitochondrial defense systems. It not only occurs in aging and age-related disorders, but also in cancer [5]. The by-products of lipid peroxidation can induce carcinogenesis. Mutations in complex I subunit dehydrogenase subunit 6 (ND6) increase the metastatic potential by producing excessive ROS, whereas an ND5 mutation enhances tumorigenesis by oxidative stress and Akt (also known as protein kinase B) activation [46,47]. Recent studies have also shown that increased mitochondrial fission is a pro-tumorigenic phenotype [44].

Cytochrome c oxidase (CcO), the terminal oxidase of the mitochondrial electron transport chain, is a highly regulated enzyme that is involved in mitochondrial oxidative metabolism and ATP synthesis. CcO dysfunction shows a consistently positive association with increased mitochondrial ROS production and cellular toxicity [43].

Mitochondrial membrane permeabilization (MMP) is an important event in pathological cell death induced by ischemia/reperfusion, xenobiotic intoxication, neurodegenerative disease, and viral infection. Inhibition of MMP constitutes an important strategy for the pharmaceutical prevention of unwarranted cell death. In contrast, the induction of MMP in tumor cells constitutes the goal of anticancer chemotherapy [30].

In cancer cells, quality control and biogenesis are often upregulated in mitochondria. Several pathologies and adverse environmental conditions disrupt mitochondrial function in multiple ways, such as MtDNA mutations, deletions, or impaired DNA replication. A few cancers produce oncogenic metabolites via mutations in nuclear-encoded mitochondrial tricarboxylic acid (TCA) cycle enzymes; in contrast, there is negative selection for pathogenic mitochondrial genome mutations [48].

### 2.2. Endothelial Dysfunction

Alterations in endothelial cells and vasculature play an important role in the pathogenesis of human vascular diseases, such as coronary heart disease, peripheral vascular disease, stroke, venous thrombosis, diabetes, tumor growth, and metastasis [20]. Endothelial dysfunction includes impaired vasodilation with decreased vascular repair capacity, increased oxidative stress, uric acid formation, increased lipid peroxide radicals, high levels of nitrotyrosine, enhanced procoagulant phenotype, enhanced proinflammatory phenotype, and increased endothelial microparticles and circulating endothelial cells [20]. Elevation of circulating endothelial cells (CECs) and reduction of endothelial progenitor cells (EPCs) are potential diagnostic biomarkers for endothelial dysfunction and they have been described in different kinds of cardiovascular diseases. CECs are mature cells that are shed from blood vessels and are rare in normally present but increased in endothelial dysfunction. The circulating endothelial progenitor cells, which arise from the bone marrow, are non-differentiated and immature endothelial cells, and contribute to the repair and renewal of damaged endothelium [49]. One study concluded that the CEC level is a more sensitive marker for vascular damage compared to the EPC level, which might be increased secondarily as a repair mechanism due to more severe vascular damage [50].

In the pathogenesis of atherosclerosis, multiple stimuli, such as oxidized low-density lipoproteins (LDLs), high glucose level, uric acid, and homocysteine, damage the integrity of the vascular endothelium, cause vessel leakage, increase leukocyte adhesion to the clammy endothelium, induce VSCM contraction, lead to endothelial nitric oxide synthase (eNOS) uncoupling, and decrease NO production [51].

**Figure 2:**
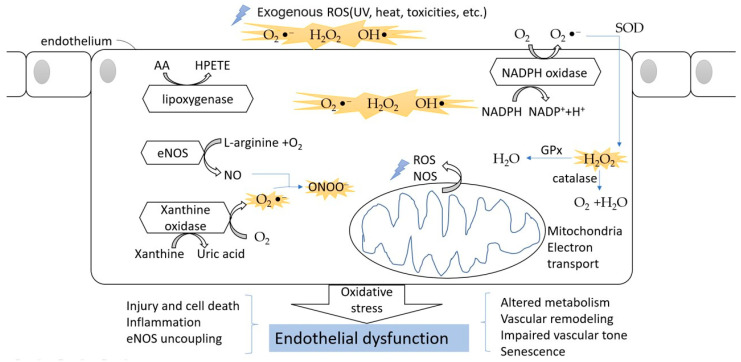
The oxidative stress in endothelial cells. The ROS can divide between an exogenous source and internal source. In the endothelium, during the processes of NO formation, uric acid generation, AA metabolism, and NADPH oxidation, ROS are released into the cytoplasm. The oxidative stress finally leads to endothelial dysfunction and affects normal physical function.

Several studies showed an impairment of endothelial function in both macro- and microvascular complications due to hyperglycemia in both animal models and human participants [52,53,54]. Hyperglycemia results in oxidative stress via an increase of the production of ROS and RNS, and affects vascular homeostasis by impairing vasorelaxation and increasing vasoconstriction, eventually inducing endothelial dysfunction. Advanced glycation end products (AGEs) also induce decreased NO synthesis, eNOS expression, and increased ET-1 expression, leading to endothelial dysfunction [55,56]. The oxidative stress in endothelial cells is shown in Figure 2.

Under the situation of endothelial dysfunction, inflammatory and procoagulant biomarkers, such as IL-6, TNF-α, PAI-1, D-dimer, and vWF, are increased in diabetic patients with micro- and macrovascular complications, including cardiovascular disease or nephropathy [54]. These proinflammatory cytokines are also important for angiogenesis and migration of endothelial cells [57]. It is interesting that TNF-α regulates both pro- and antiangiogenic properties and interacts with two distinct transmembrane receptors: tumor necrosis factor receptor 1 (TNFR1) and tumor necrosis factor receptor 2 (TNFR2). TNFR1 is related to differentiation, cell death, and apoptosis, while TNFR2 cell maintains cell survival and proliferation. A recent report showed that human EPCs are immunosuppressive and this effect was TNF-α/TNFR2 dependent [58,59].

Endothelial dysfunction of vessels was characterized by increased blood levels of vascular endothelial growth factor (VEGF), endothelin-1 (ET-1), P-selectin (PSel), homocysteine (Hcys), nitrites (NO_2_), and cyclic guanosine monophosphate (cGMP), as well as decreased PGI2 values. These indices of increasing VEGF and ET-1 and decreasing PGI2 were observed in most lung cancer cases. Disturbances of vascular endothelial function were associated with the patient’s age, disease duration, morphological form, and lung cancer stage [60]. Angiotensin II (Ang II), as a powerful vasoconstrictor for controlling blood pressure and vascular remodeling, has also been shown to have important inflammatory and oxidative actions in the endothelium [61,62]. Ang II promotes apoptosis in endothelial cells by generating ROS, increasing NADPH oxidase (Nox) activity in endothelial cells, and enhancing superoxide production via AT_1_R and AT_2_R [63,64].

Endothelial cells are coupled with malignant tumor cells in almost every stage of the metastatic steps, including infiltration of cancer cells into the nearby tissue; endothelial transmigration, also calls “intravasation”; survival in the blood stream; and extravasation followed by colonization of the target organ. Dysfunctional blood vessels within the tumor are heterogeneous and highly permeable, resulting from the activity of factors, such as hypoxia and chronic growth factor stimulation [65]. Cancer metastasis and secondary tumor initiation largely depend on circulating tumor cells and vascular endothelial cell interactions. Endothelial glycocalyx (GCX) dysfunction may play a significant role in this process [65,66]. One study showed that microvascular endothelial dysfunction, as defined by a reactive hyperemia peripheral arterial tonometry index ≤2.0, was associated with an increase of over two times the risk of solid tumor cancer. Besides its known ability to predict cardiovascular disease, microvascular endothelial dysfunction may be a useful index for solid tumor cancer prediction [67].

### 2.3. Salvia miltiorrhiza and Mitochondrial Oxidative Stress

Various oxidases, including xanthine oxidase, uncoupled NOS, cytochrome P450 enzymes, and mitochondrial and NADPH oxidases (Nox), produce superoxide, which is toxic via univalent reduction [68,69]. Superoxide dismutases (SODs) are the major antioxidant enzymes that degrade superoxide to the more stable ROS, H_2_O_2_, which is then converted to water and oxygen by either catalase or glutathione peroxidase (GPx) [70]. There are three isoforms of SOD in mammals: cytoplasmic Cu/ZnSOD (SOD1), mitochondrial MnSOD (SOD2), and extracellular Cu/ZnSOD (SOD3) [71].

SM may reduce ROS production by inhibiting oxidases, reducing the production of superoxide, inhibiting the oxidative modification of low-density lipoproteins (LDLs), and ameliorating mitochondrial oxidative stress. SM also increases the activities of catalase, manganese SOD, GPx, and eNOS [72]. In a previous study, SM hydrophilic extract could reverse the induction of vascular endothelial growth factor (VEGF) expression by high glucose levels through mitigation of mitochondrial oxidative stress [73].

The Kelch-like ECH-associated protein 1 (Keap1)-nuclear factor erythroid 2-related factor 2 (Nrf2) system is central for mammalian cyto-protection against electrophilic and oxidative stress [74]. In acetaminophen-induced hepatocyte injury, one study found that the component salvianolic acid C exhibits a protective effect by ameliorating inflammatory response, caspase-mediated anti-apoptotic effects, and mitochondrial oxidative stress through inhibition of the Keap1/Nrf2/heme oxygenase-1 (HO-1) signaling axis [75].

Malondialdehyde (MDA) is one of the final products of polyunsaturated fatty acid peroxidation in the cells. An increasing MDA level is recognized as a relevant biomarker of oxidative stress with free radical overproduction [76,77]. In neurological areas, glutamate excitotoxicity is related to several diseases, including cerebral ischemia and neurodegenerative diseases. One study [78] revealed that Tan IIA could suppress glutamate-induced oxidative stress by reducing ROS levels and MDA, and by increasing the activities of SOD and catalase. Tan IIA prevents glutamate-induced mitochondrial dysfunction by enhancing the mitochondrial membrane potential and ATP content, and by decreasing the mitochondrial protein carbonyl content. Furthermore, Tan IIA can suppress glutamate-induced apoptosis through regulation of apoptosis-related protein expression, including an elevation of B cell lymphoma 2 (Bcl-2) protein levels, reduction of Bcl-2-associated X (Bax) and cleaved caspase-3 levels, and suppression of Jun N-terminal kinase (JNK)1/2, and furthermore, p38 mitogen-activated protein kinase (MAPK) activation [79].

Tan IIA administration resulted in a significant decrease in the mitochondrial fusion proteins, mitofusin (Mfn) 1/2 and Optic atrophy 1 (Opa1), as well as an increase in the fission protein dynamin-related protein 1 (Drp1) in human osteosarcoma cells [80].

### 2.4. Salvia miltiorrhiza in Endothelium Dysfunction

Dihydrotanshinone, cryptotanshinone, Tan I, and Tan IIA, as new acetylcholinesterase (AChE) inhibitors, have the potential to penetrate the blood–brain barrier and may be used to treat Alzheimer’s disease [81].

Dihydrotanshinone exerts a vasorelaxant effect by inhibiting Ca^2+^ influx in VSMCs, and it is independent of pathways involving the endothelium, muscarinic receptors, beta-adrenoceptors, adenylyl cyclase, and guanylyl cyclase [82].

Cryptotanshinone (CTS) possesses anti-inflammatory properties by suppressing the TNF-α-induced increase in endothelial permeability, monocyte adhesion, intercellular cell adhesion molecule-1 (ICAM-1), vascular cell adhesion molecule-1 (VCAM-1), and monocyte chemoattractant protein-1 (MCP-1), and restoring NO production [83]. CTS significantly inhibited sodium-nitroprusside (SNP)-induced cell toxicity and the generation of ROS and RNS, and improved the mitochondrial membrane potential (MMP) in neuro-2a (N2a) cells. CTS significantly inhibited SNP-induced peroxidation of lipids and proteins and the expression of glutamate-cysteine ligase catalytic subunit (Gclc) mRNA. CTS elevated Akt and cyclic AMP response element-binding protein and further blocked SNP-induced activation of nuclear factor-kappa B (NF-κB), extracellular signal-regulated kinase (ERK)1/2, and JNK/MAPK pathways. Additionally, the increase in the mitochondrial Bax/Bcl-2 ratio, activation of cytosolic procaspase-3, and release of cytochrome c from mitochondria to the cytosol were significantly reduced by CTS [84].

Tan IIA reduced intracellular oxidative stress and increased NO generation by restoring high glucose-induced eNOS uncoupling by targeting the NADPH oxidase, heat shock protein 90 (HSP90) [85], GTP cyclohydrolase-1 (GTPCH1) [86], dihydrofolate reductase (DHFR) [87], and Phosphoinositide 3-kinases (PI3K) pathways [88,89]. Endothelial injury and subsequent atherogenic events may be provoked by chronic oxidative stressors like H_2_O_2_ and methylglyoxal. Tan IIA has the potential to stabilize atherosclerotic plaques by inhibiting LDL oxidation, monocyte adhesion to the endothelium, smooth muscle cell migration and proliferation, macrophage cholesterol accumulation, proinflammatory cytokine expression, and platelet aggregation [90]. In vivo, the atherosclerotic change region decreases by 3.5 times after Tan IIA treatment. Intracellular chloride channel 1 (CLIC1) is involved in the oxidative stress and inflammatory process. Tan IIA reduced MDA production, increased SOD activity, decreased TNF-α and IL-6 levels, and suppressed the expression of CLIC1, ICAM-1, and VCAM-1 in atherosclerotic mice. In vitro, the antioxidative and anti-inflammatory effects of Tan IIA were dose dependent, further confirming this result. Moreover, CLIC1 depletion abolished the Tan IIA-mediated decrease in ROS and MDA production in human umbilical vein endothelial cells (HUVECs). Additionally, Tan IIA inhibited both CLIC1 membrane translocation and the chloride ion concentration [91].

The endothelial protective effects of Tan IIA derivatives enhanced efficacy against H_2_O_2_-induced injury via Nrf2 activation and excellent hydrophilic activity [92]. CD31, also known as platelet endothelial cell adhesion molecule (PECAM-1) [93], was suppressed in Tan IIA-treated xenografts, indicating antineovascularization. Tan IIA facilitates Bcl-2 translocation to the mitochondrial outer membrane, prevents mitochondrial permeability transition pore opening, decreases cytochrome c release, prevents caspase-3 activation, and restrains apoptosis [94].

Ursolic acid, an aqueous extract of SM, also reduced the expression of the NADPH oxidase subunit Nox4 and suppressed the production of ROS in human endothelial cells [95]. The vasodilatation mechanisms of SM aqueous extract and salvianolic acid B were produced by the inhibition of Ca^2+^ influx in VSMCs. The opening of K^+^ channels had a minor contribution to their effects, but endothelium-dependent mechanisms were not involved [96].

Salvianolic acid A (SalA) treatment inhibited the toll-like receptor 4 and nuclear factor kappa B pathway, and prompted a lowering of proinflammatory mediators including IL-1β, IL-6, TNF-α, ICAM-1, and VCAM-1. In addition, SalA treatment significantly decreased oxidative stress by increasing antioxidant enzyme activity, upregulating the nuclear factor erythroid 2-related factor 2/heme oxygenase-1 pathway, and downregulating the expression of p47phox and p22phox in vivo. p47phox, known as neutrophil cytosol factor 1, relates to activation of NADPH oxidase and is required for atherosclerosis lesion progression in ApoE^−/−^ mice [97,98]. p22phox, also known as the human neutrophil cytochrome b light chain, is an essential component of the membrane-associated enzyme phagocyte NADPH-oxidase and exists in endothelial and vascular smooth muscle cells. Furthermore, SalA suppressed oxidized LDL-induced expression of lectin-like oxidized LDL receptor-1, the phosphorylation of nuclear factor kappa B (p65), ICAM-1, and VCAM-1, and inhibited NADPH oxidase subunit 4-mediated ROS generation in HUVECs [99]. SalA protects HUVECs against tert-butyl hydroperoxide-induced oxidative injury via a mitochondria-dependent pathway [100]. SalA inhibits endothelial dysfunction and vascular remodeling in spontaneously hypertensive rats. Therefore, Sal A could be a potential drug therapy to prevent further targeted organ damage induced by vascular remodeling [101].

Salvianolic acid B (SalB) prevents oxidative stress-induced endothelial dysfunction by downregulating Nox-4, eNOS, and nicotinamide adenine dinucleotide phosphate (NADPH)-oxidase expression. During apoptosis, mitochondria take over multiple apoptotic signals. The Bcl-2 family of proteins regulate the promotion or inhibition of apoptosis [102]. Bax and Bak, as proapoptotic members, result in the release of cytochrome c from mitochondria [103], whereas Bcl-2 and Bcl-extra large (Bcl-xL) are the common antiapoptotic proteins that promote cell survival [104]. SalB decreased the Bax/Bcl-xL ratio and caspase-3 activation after H_2_O_2_ induction. One study revealed that activation of the mTOR/p70S6K/4EBP1 pathway is required for both SalB-mediated angiogenic and protective effects against oxidative stress-induced cell injury in human bone marrow-derived endothelial progenitor cells (BM-EPCs). SalB suppress mitogen-activated protein kinase 3 and 6 (MKK3/6)-p38 MAPK-ATF2 and ERK1/2 signaling pathways, reduces intracellular ROS levels and apoptosis, and further protects BM-EPCs against oxidative stress-related cell injury [105]. Sal B decreased the Ang II-induced elevation of arterial systolic blood pressure in mice, increased the impaired endothelium-dependent relaxation, and attenuated the endothelium-dependent over-contractions in both the aorta and renal arteries of Ang II-infused mice. Furthermore, Sal B treatment stabilized the elevating AT1 receptors, NADPH oxidase subunits (Nox-2 and Nox-4), and nitrotyrosine in the arteries of Ang II-infused mice or in Ang II-treated HUVECs [106] (Table 1).

## 3. Conclusions

Through this review, we found that SM has antioxidative, anti-inflammatory, and antithrombotic effects. SM can decrease ROS formation in the mitochondria, preventing endothelial cell dysfunction. Endothelial dysfunction may be related to many cardiovascular and cerebrovascular diseases, such as stroke, acute myocardial infarction, peripheral vascular disease, and Alzheimer’s disease. Both lipophilic and hydrophilic components of SM can protect the endothelium against mitochondrial oxidative stress. More research is needed to discover the mechanism of SM in preventing oxidative stress in the endothelium.

## Figures and Tables

**Table 1:** The antioxidative mechanisms of *Salvia miltiorrhiza*.

Bioactive Compounds	Preventing Mechanism	Reference
SM	↓ ROS, O_2_^•−^ ⊝ oxidases, oxLDL, and ameliorating mitochondrial oxidative stress. ↑ catalase, SOD, GPx, and coupled eNOS	[72]
SM hydrophilic extract	⊝ VEGF expression, Ca^2+^ influx in VSMC ⊕ vasorelaxant ameliorating mitochondrial oxidative stress	[73]
Salvianolic acid A	↑ SOD, Nrf2/HO-1 pathway ↓ IL-1β, IL-6, TNF-α, ICAM-1, VCAM-1 ⊝ TLR4/NF-κB pathway, oxLDL, p47phox and p22phox, LOX-1, NF-κB p65 phosphorylation, Nox4	[99]
Salvianolic acid B	↓ Nox4, eNOS ⊝ Ca^2+^ influx in VSMC, Bax/Bcl-xL ratio, caspase-3, MKK3/6-p38 MAPK-ATF2 and ERK1/2 signaling pathways ⊕ vasorelaxant, mTOR/p70S6K/4EBP1 pathways	[96,105]
Salvianolic acid C	↓ Inflammation, oxidative Stress, and apoptosis ⊝ Keap1/Nrf2/HO-1 signaling axis	[75]
Ursolic acid	↓ Nox4, ROS	[95]
Tanshinone I	⊝ AChE	[81]
Tanshinone IIA	↑ Bcl-2, mitochondrial membrane potential and ATP, SOD, catalase, NO, ratio of BH4 to BH2, Drp1 ↓ ROS, Bax, caspase-3, eNOS uncoupling, atherosclerotic lesion, MDA, Mfn1/2 and Opa1, cytochrome c release ⊕ HSP90, GTPCH1, DHFR, Nrf2, 14-3-3η ⊝ JNK, p38 MAPK, AChE, O_2_^•−^, Nox4, PI3K, LDL oxidation, monocyte adhesion, SMC migration and proliferation, macrophage cholesterol accumulation, proinflammatory cytokine expression, platelet aggregation, CLIC1, ICAM-1, VCAM-1, CD31, mitochondrial permeability transition pore opening	[78,79,80,81,88,91,92,94]
Dihydrotanshinone	⊝ Ca^2+^ influx in VSMC, AChE	[81,82]
Cryptotanshinone	⊝ AChE, TNF-α ↓ endothelial permeability, monocyte adhesion, ICAM-1, VCAM-1 and MCP-1 ⊕ NO	[81,83]

↑: increase; ↓: decrease; ⊝: inhibit; ⊕: promote SM, *Salvia miltiorrhiza*; ROS, reactive oxygen species; oxLDL, oxidized low-density lipoprotein; SOD, superoxide dismutase; GPx, glutathione peroxidase; VEGF, vascular endothelial growth factor; AChE, Acetylcholinesterase; BH4, tetrahydrobiopterin; BH2, 7,8-dihydrobiopterin; HSP90, heat-shock protein of 90 kDa; GTPCH1, GTP cyclohydrolase-1; DHFR, dihydrofolate reductase; VSMC, vascular smooth muscle cells; IL-1β, interleukin-1β; IL-6, interleukin-6; TNF-α, tumor necrosis factor-α; ICAM-1, intercellular cell adhesion molecule-1; VCAM-1, vascular cell adhesion molecule-1; TLR4/NF-κB, toll-like receptor 4/nuclear factor kappa B; LOX-1, Lectin-like oxidized low-density lipoprotein receptor-1; NO, nitric oxide; O_2_^•−^, superoxide; SMC, smooth muscle cell; MDA, malondialdehyde; CLIC1, Intracellular chloride channel 1; Nrf2, nuclear fac-tor (erythroid-derived 2)-like-2 factor; Drp1, dynamin-related protein 1; Opa1, Optic atrophy 1; Mfn1/2, Mitofusin 1/2.

## Data Availability

Not applicable.
